# KIR2DL2 combined with HLA-C1 confers risk of hepatitis C virus-related hepatocellular carcinoma in younger patients

**DOI:** 10.18632/oncotarget.24752

**Published:** 2018-04-13

**Authors:** Hiromi Saito, Takeji Umemura, Satoru Joshita, Tomoo Yamazaki, Naoyuki Fujimori, Takefumi Kimura, Michiharu Komatsu, Akihiro Matsumoto, Eiji Tanaka, Masao Ota

**Affiliations:** ^1^ Department of Medicine, Division of Hepatology and Gastroenterology, Shinshu University School of Medicine, Matsumoto, Japan; ^2^ Research Center for Next Generation Medicine, Shinshu University, Matsumoto, Japan

**Keywords:** killer cell immunoglobulin-like receptor, human leukocyte antigen, hepatitis C virus, hepatocellular carcinoma, MICA

## Abstract

Killer cell immunoglobulin-like receptors (KIRs) are involved in the activation and inhibition of natural killer cells. Although combinations of KIRs and HLA have been associated with spontaneous and treatment-induced clearance of hepatitis C virus (HCV) infection, their roles are not fully understood in the context of hepatocellular carcinoma (HCC) development. We enrolled 787 consecutive patients with chronic HCV infection, which included 174 cases of HCC, and 325 healthy subjects to clarify the involvement of HLA-Bw and C, KIRs, and major histocompatibility complex class I chain-related gene A (MICA) gene polymorphisms (rs2596542 and rs1051792) in chronic HCV infection and HCV-related HCC. We observed a significant association with chronic hepatitis C susceptibility for HLA-Bw4 (*P* = 0.00012; odds ratio [OR] = 1.66) and significant protective associations for HLA-C2 and KIR2DL1-HLA-C2 (both *P* = 0.00099; OR = 0.57). When HCC patients were stratified into younger (<65 years) and older (≥65 years) groups, the frequencies of KIR2DL2-HLA-C1 and KIR2DS2-HLA-C1 (*P* = 0.008; OR = 2.89 and *P* = 0.015; OR = 2.79, respectively) as well as rs2596542 and rs1051792 (*P* = 0.020; OR = 2.17 and *P* = 0.038; OR = 2.01, respectively) were significantly higher in younger patients. KIR2DL2-HLA-C1 (OR = 2.75; 95% confidence interval: 1.21-6.21, *P* = 0.015) and rs1051792 (OR = 2.48; 95% confidence interval: 1.23-4.98, *P* = 0.011) were independently associated with HCC development in younger patients. These results suggest that KIR2DL2-HLA-C1 and rs1051792 may represent molecular biomarkers to identify early onset HCV-related HCC.

## INTRODUCTION

Hepatitis C virus (HCV) infection is a major cause of chronic liver disease, with over half of patients with acute HCV infection developing chronic hepatitis and eventually liver cirrhosis and/or hepatocellular carcinoma (HCC) [1]. In Japan, HCC is one of the primary causes of cancer-related death, among which HCV-related HCC accounts for 60-70% of HCC cases nationwide [2]. The mechanisms involved in the progression of HCV infection to HCC have been related to viral, environmental, and host factors [3].

Natural killer (NK) cells respond to tumor cells and virally infected cells and kill them through the release of cytolytic granules or various chemokines and pro-inflammatory cytokines [4]. NK cells express multiple activation and inhibitory receptors to recognize changes in target cell state, and NK cell activity is controlled by a delicate balance of stimulation and inhibitory signals [5]. The recognition of target cells by NK cells is determined by the integration of receptor signaling. Activation receptors on NK cells mainly recognize pathogen-derived, stress-induced, and tumor-specific ligands [6], while most inhibitory receptors interact specifically with human leukocyte antigen (HLA) class I molecules in humans [7]. The common ligands of NK cell receptors are HLA class I molecules (HLA-A,-B,-C,-E, and -G) and major histocompatibility complex (MHC) class I-like molecules (MHC class I chain-related molecules A and B; MICA and MICB) [8]. NK cell surface receptors include the killer cell immunoglobulin-like receptor (KIR) family, which bind HLA-A,-B,-C, or -G, in addition to lectin-like molecules, inhibitory CD94-NKG2A, and activating CD94-NKG2C, which interact with HLA-E. Another NK cell-activating lectin-like receptor, NKG2D, recognizes multiple stress-inducible molecules, MICA, MICB, and the cytomegalovirus UL16-binding protein [9]. Thus, immunosurveillance for malignant cells is mainly mediated by the recognition of HLA class I molecules by KIRs and such NK cell-activating lectin-like receptors as NKG2D. In line with this model, the downregulation of MHC class I molecules on target cells promotes the activation of NK cells and their cytotoxic activity. CD94/NKG2 heterodimers and KIRs are expressed by most NK cells and a minority of T cells. The inhibitory KIR2DL1 recognizes HLA-C group 2 (HLA-C2) allotypes that share lysine at position 80, while KIR2DL2 and KIR2DL3 are specific for HLA-C group 1 (HLA-C1) allotypes having asparagine at position 80 with different affinity [10]. KIR2DL2 and KIR2DL3 also recognize HLA-B^*^46:01 acquiring the C1 epitope by gene conversion [11]. Furthermore, KIR3DL1 binds with high affinity to HLA-Bw4 molecules containing isoleucine at position 80 (Bw4[80I]) as compared with threonine (Bw4[80T]) [12].

NK cells have been implicated in the pathogenesis of HCV infection and HCC [13], specifically in the destruction of HCV-infected cells. The loss of NK cell activity is correlated with the progression of HCV infection to HCC [14]. Receptor-ligand combinations of KIR and HLA have been associated with the natural clearance of HCV infection and anti-viral therapy outcome in patients with chronic hepatitis C [15–19]. Although several studies have described the relationship between KIR-HLA and HCV-related HCC [20, 21], the exact impact and role of KIR-HLA remains unknown in the Japanese population. Moreover, a recent genome-wide association study revealed that single nucleotide polymorphisms (SNPs) in the MICA gene imparted susceptibility to HCV-related HCC in Japan [22].

The present study sought to clarify the relationships of HLA, KIR, KIR-HLA, and MICA SNPs with chronic HCV infection and the development of HCC in the Japanese population.

## RESULTS

### Patient characteristics

The demographic, virological, and clinical characteristics of our cohort are summarized in Table 1. Among the 787 patients, 174 (22.1%) were diagnosed as having HCC. These patients had a significantly higher male ratio compared with controls. Age, bilirubin, ALT, and AFP were significantly higher and albumin, platelet count, and PT% were significantly lower in patients with HCC than in those without.

**Table 1:** Demographic and clinical characteristics of patients

Characteristic	Total (n = 787)	Patients with HCC (n = 174)	Patients without HCC (n = 613)	*P* value
Age, y	68 (43-82)	70 (52-82)	67 (41-82)	0.011
Male, n (%)	365 (46.4)	104 (59.8)	261 (42.6)	<0.001
Albumin, g/dL	4.2 (3.4-4.7)	3.8 (3.0-4.5)	4.3 (3.6-4.8)	<0.001
Bilirubin, mg/dL	0.8 (0.4-1.6)	0.9 (0.5-1.8)	0.8 (0.4-1.5)	0.006
ALT, IU/L	44 (17-178)	56 (19-194)	39 (16-173)	<0.001
Platelet count, ×10^9^/L	15.8 (6.7-25.6)	10.6 (4.9-21.6)	16.8 (9.1-26.9)	<0.001
PT%	93.7 (69.4-116.3)	84.6 (59.3-110.5)	97.8 (74.6-118.1)	<0.001
AFP, ng/mL	4.4 (1.5-110.6)	17.7 (2.3-465.1)	3.6 (1.4-32.8)	<0.001
HCV RNA, log IU/mL	6.5 (4.2-7.4)	6.5 (3.4-7.4)	6.5 (4.3-7.4)	0.219
HCV genotype 1, n (%)	622 (79.0)	144 (82.8)	478 (78.0)	0.194

Parameters are presented as the median (5^th^-95^th^ percentiles) for continuous variables and total number (%) for categorical variables. Abbreviations: HCC, hepatocellular carcinoma; ALT, alanine aminotransferase; PT, prothrombin time; AFP, α fetoprotein; HCV, hepatitis C virus.

### HLA and KIR genotyping in patients with chronic HCV infection and controls

**Figure 1:**
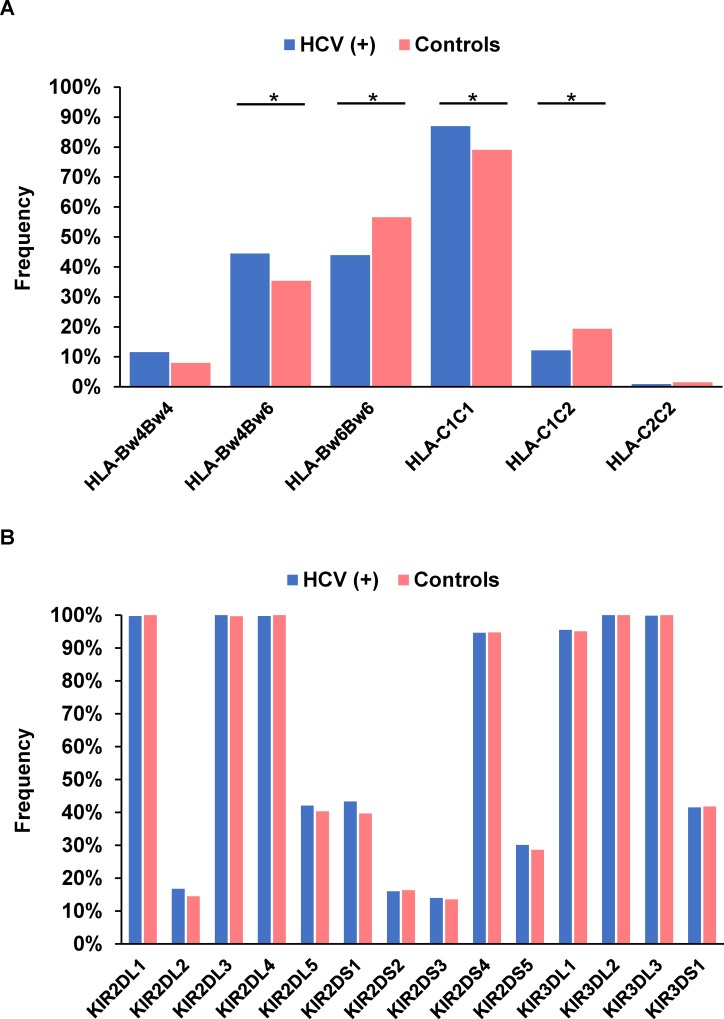
Frequency of HLA-Bw and -C **(A)** and each killer cell immunoglobulin-like receptor gene **(B)** in 787 patients with chronic hepatitis C and 325 healthy subjects. ^*^P < 0.05.

Among HLA-Bw and -C alleles, the frequency of HLA-Bw4Bw6 in patients with chronic HCV infection was significantly higher than that in healthy subjects (44.5% versus 35.4%; *P* = 0.0052; OR = 1.46) (Figure 1A) (Table 2). Conversely, the HLA-Bw6 homozygote had a significantly lower frequency in HCV-infected patients (44.0% versus 56.6%; *P* = 0.00012; OR = 0.60). The frequency of HLA-Bw4 was significantly higher in patients with chronic hepatitis C than in controls (56.0% versus 43.4%; *P* = 0.00012; OR = 1.66), as were those of HLA-Bw4(80I) (38.8% versus 32.0%; *P* = 0.034; OR = 1.34) and HLA-Bw4(80T) (21.3% versus 13.2%; *P* = 0.0017; OR = 1.78). The presence of one copy of HLA-C1 (HLA-C1C2) was significantly associated with controls (*P* = 0.0018; OR = 0.58), while that of two copies (HLA-C1C1) was found more frequently in patients with chronic hepatitis C (86.9% versus 79.1%; *P* = 0.00099; OR = 1.76). Hence, higher HLA-C1 copy number was associated with more frequent chronic HCV infection. Lastly, HLA-C2 was significantly less frequent in patients with chronic HCV infection (13.1% versus 20.9%; *P* = 0.00099; OR = 0.57). With respect to KIR genes, there were no significant differences between HCV-infected patients and healthy controls (Figure 1B).

**Table 2:** Frequency of HLA, KIR genes, and KIR-HLA combinations in patients with chronic hepatitis c and healthy controls

Genetic factor	HCV-positive n = 787	Controls n = 325	OR	95% CI	*P* value
HLA-Bw4	441 (56.0)	141 (43.3)	1.66	1.28-2.16	0.00012
HLA-Bw4(80I)	305 (38.8)	104 (32.0)	1.34	1.02-1.77	0.034
HLA-Bw4(80T)	168 (21.3)	43 (13.2)	1.78	1.24-2.56	0.0017
HLA-Bw4Bw4	91 (11.6)	26 (8.0)	1.50	0.95-2.37	0.078
HLA-Bw4Bw6	350 (44.5)	115 (35.4)	1.46	1.12-1.91	0.0052
HLA-Bw6Bw6	346 (44.0)	184 (56.6)	0.60	0.46-0.78	0.00012
HLA-C1	780 (99.1)	320 (98.5)	1.74	0.55-5.53	0.34
HLA-C2	103 (13.1)	68 (20.9)	0.57	0.41-0.80	0.00099
HLA-C1C1	684 (86.9)	257 (79.1)	1.76	1.25-2.46	0.00099
HLA-C1C2	96 (12.2)	63 (19.4)	0.58	0.41-0.82	0.0018
HLA-C2C2	7 (0.9)	5 (1.5)	0.57	0.18-1.82	0.34
2DL1+HLA-C2	103 (13.1)	68 (20.9)	0.57	0.41-0.80	0.00099
2DS1+HLA-C2	40 (5.1)	26 (8.0)	0.62	0.37-1.03	0.061
3DL1+HLA-Bw4	425 (54.0)	135 (41.5)	1.65	1.27-2.15	0.00016
3DL1+HLA-Bw4(80I)	296 (37.6)	99 (30.5)	1.38	1.04-1.82	0.023
3DL1+HLA-Bw4(80T)	161 (20.5)	42 (12.9)	1.73	1.20-2.50	0.0031

Parameters are presented as total number (%).

Abbreviations: HLA, human leukocyte antigen; KIR, killer cell immunoglobulin-like receptor; HCV, hepatitis C virus; OR, odd ratio; CI, confidence interval.

We next analyzed combinations of activation/inhibitory KIRs and their ligands for possible relationships with HCV infection. The frequency of the KIR2DL1-HLA-C2 combination in patients with chronic hepatitis C was significantly lower than that in controls (13.1% versus 20.9%; *P* = 0.00099; OR = 0.57). In contrast, HCV-infected patients had a significantly higher frequency of KIR3DL1-HLA-Bw4 (54.0% versus 41.5%; *P* = 0.00016; OR = 1.65). It was noteworthy that KIR3DL1-HLA-Bw4(80I) and KIR3DL1-HLA-Bw4(80T) were significantly more common in HCV-infected patients (37.6% versus 30.5%; *P* = 0.023; OR = 1.37 and 20.5% versus 12.9%; *P* = 0.0031; OR = 1.73, respectively).

### HLA and KIR genotyping in patients with or without HCC

**Figure 2:**
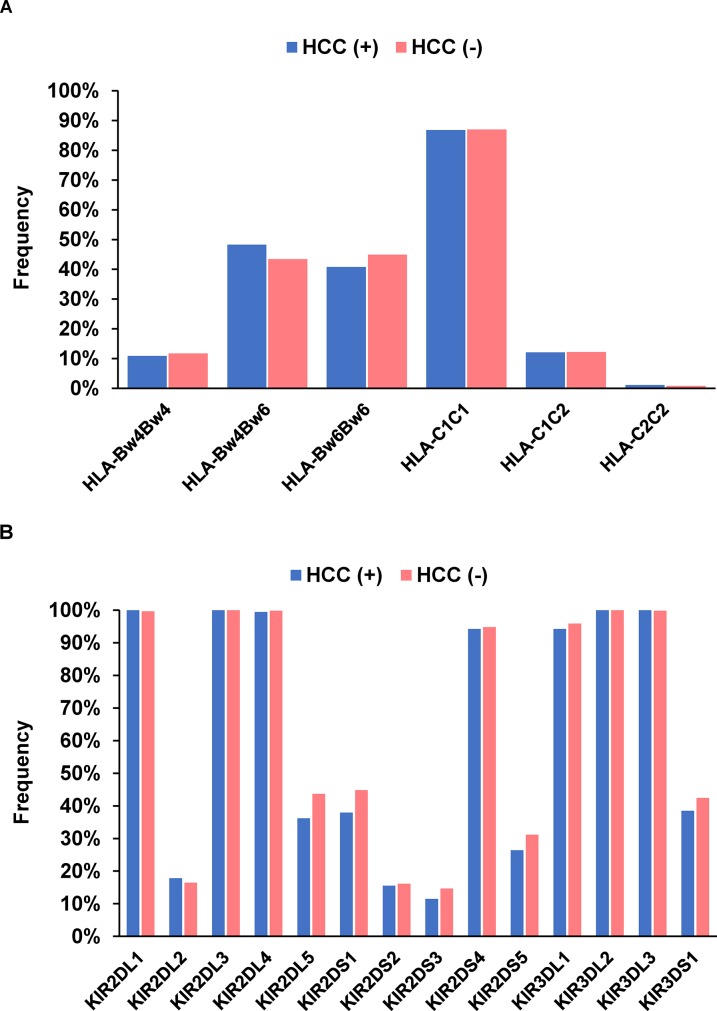
Frequency of HLA-Bw and -C **(A)** and each killer cell immunoglobulin-like receptor gene **(B)** in 174 patients with hepatocellular carcinoma and 613 patients without.

To clarify the effect of HLA alleles and KIR genes on HCC development, their genotype frequencies were compared between HCV-infected patients with and without HCC. No specific HLA alleles (Figure 2A) or KIR genes (Figure 2B) were detected, nor were there any significant relationships involving KIR-HLA combinations.

**Figure 3:**
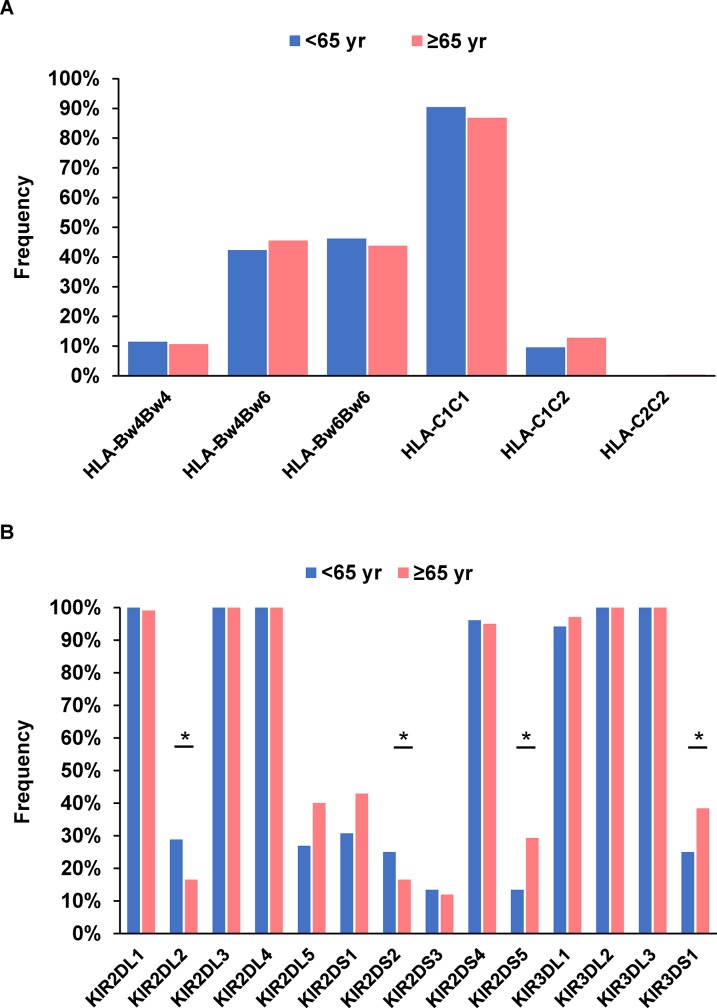
Frequency of HLA-Bw and -C **(A)** and each killer cell immunoglobulin-like receptor gene **(B)** in 52 younger (<65 years) patients and 122 older (≥65 years) patients with hepatocellular carcinoma. ^*^P < 0.05.

Next, we stratified patients with chronic hepatitis C into younger (<65 years) and older (≥65 years) groups to examine the clinical characteristics and associations of HLA and KIR genes with HCC development according to age (Figure 3A and 3B). Younger patients with HCC had a significantly higher male ratio, significantly higher ALT, and significantly lower PT% (Table 3). The frequencies of the HLA-Bw4 and HLA-C1 alleles were comparable between the groups (Table 4). With respect to KIR genes, the frequencies of KIR2DL2 and KIR2DS2 were significantly higher in younger HCC patients (28.8% versus 13.1%; *P* = 0.013; OR = 2.69 and 25.0% versus 11.5%; P = 0.024; OR = 2.57, respectively) (Table 4). Conversely, younger patients had significantly lower frequencies of KIR2DS5 (13.5% versus 32.0%; *P* = 0.018; OR = 0.33) and KIR3DS1 (25.0% versus 44.3%; *P* = 0.017; OR = 0.42). We then evaluated the influence of KIR-HLA combinations on HCC development in younger patients and witnessed the combinations of KIR2DL2-HLA-C1 and KIR2DS2-HLA-C1 to be significantly associated with younger HCC patients versus older patients (28.8% versus 12.3%; *P* = 0.008; OR = 2.89 and 25.0% versus 10.7%; *P* = 0.015; OR = 2.79, respectively). No other KIR or KIR-HLA combinations differed between the groups.

**Table 3:** Demographic and clinical characteristics of patients with HCC stratified by age

Characteristic	<65 years (n = 52)	≥65 years (n = 122)	*P* value
Age, y	60 (46-64)	72 (65-83)	<0.001
Male, n (%)	37 (71.2)	67 (54.9)	0.046
Albumin, g/dL	3.8 (3.1-4.5)	3.8 (2.8-4.5)	0.909
Bilirubin, mg/dL	0.9 (0.4-2.9)	0.8 (0.5-1.6)	0.211
ALT, IU/L	62 (23-264)	53 (18-154)	0.028
Platelet count, ×10^9^/L	10.1 (3.6-25.6)	10.9 (5.3-20.8)	0.317
PT%	79.0 (57.3-115.9)	86.3 (61.9-109.9)	0.012
AFP, ng/mL	25.8 (3.0-175.1)	15.6 (2.1-666.9)	0.236
HCV RNA, log IU/mL	6.6 (2.8-7.4)	6.3 (3.4-7.4)	0.291
HCV genotype 1, n (%)	39 (75.0)	105 (86.1)	0.077

Parameters are presented as the median (5^th^-95^th^ percentiles) for continuous variables and total number (%) for categorical variables.

Abbreviations: HCC, hepatocellular carcinoma; ALT, alanine aminotransferase; PT, prothrombin time; AFP, α fetoprotein; HCV, hepatitis C virus.

**Table 4:** Frequency of HLA, KIR genes and KIR-HLA combinations in patients with HCC stratified by age

Genetic factor	<65 years (n = 52)	≥65 years (n = 122)	OR	95% CI	*P* value
HLA-Bw4	28 (53.8)	75 (61.5)	0.73	0.38-1.41	0.35
HLA-C1	52 (100.0)	120 (98.4)	-	-	0.35
2DL2	15 (28.8)	16 (13.1)	2.69	1.21-5.96	0.013
2DS2	13 (25.0)	14 (11.5)	2.57	1.11-5.95	0.024
2DS5	7 (13.5)	39 (32.0)	0.33	0.14-0.80	0.011
3DS1	13 (25.0)	54 (44.3)	0.42	0.20-0.86	0.017
2DL2+HLA-C1	15 (28.8)	15 (12.3)	2.89	1.29-6.48	0.008
2DL2+HLA-C1C1	15 (28.8)	12 (9.8)	3.72	1.60-8.66	0.0015
2DS2+HLA-C1	13 (25.0)	13 (10.7)	2.79	1.19-6.55	0.015
2DS2+HLA-C1C1	13 (25.0)	10 (8.2)	3.73	1.52-9.20	0.0027

Parameters are presented as total number (%).

Abbreviations: HLA, human leukocyte antigen; KIR, killer cell immunoglobulin-like receptor; HCC, hepatocellular carcinoma; OR, odd ratio; CI, confidence interval.

Since several studies have reported that KIR-ligand copy number may influence NK cell functionality, we searched for associations between KIR2DL2 or KIR2DS2-HLA-C1 copy number and HCC development in the younger group (Table 4). HCC patients were stratified by HLA-C1 copy number into those lacking an HLA-C1 allele (HLA-C2C2), those carrying one HLA-C1 allele (HLA-C1C2), and those carrying homozygote HLA-C1 alleles (HLA-C1C1) (Table 4). Patients expressing KIR2DL2-HLA-C1 motifs were significantly more frequent in the younger group (28.8%) compared with the older group (9.8%; *P* = 0.0015; OR = 3.72). Similarly, when KIR2DS2-HLA-C1 copy number was examined, the frequency of HCC patients with homozygote C1 alleles was also higher in the younger group (25.0% versus 8.2%; *P* = 0.0027; OR = 3.73). Among chronic HCV-infected patients without HCC, no HLA alleles or KIR genes were significantly associated with younger or older groups.

### MICA SNP genotyping

We evaluated two MICA SNPs for associations with chronic hepatitis C and HCV-related HCC in our cohort. The frequencies of the rs2596542 A allele (39.1% versus 34.2%; *P* = 0.027; OR = 1.24) and rs1051792 A allele (40.3% versus 34.3%; *P* = 0.0078; OR = 1.29) in patients with chronic hepatitis C were significantly higher than those in controls, but no remarkable results were observed between patients with and without HCC. When HCC patients were stratified according to younger or older age, the frequencies of the minor A alleles at rs2596542 and rs1051792 were significantly higher in the younger HCC group (44.2% versus 32.4%; *P* = 0.035; OR = 1.66 and 52.9% versus 37.7%; *P* = 0.0087; OR = 1.85, respectively). The frequency of the AA genotype of both SNPs also differed significantly between the age groups (25.0% versus 10.7%; *P* = 0.015; OR = 2.79 and 44.2% versus 24.6%; *P* = 0.010; OR = 2.43, respectively).

### Factors independently associated with HCC in younger individuals

The results of logistic regression analysis regarding factors associated with HCC development in younger patients revealed KIR2DL2-HLAC1C1 (OR = 2.75; 95% CI: 1.21-6.21, *P* = 0.015) and rs1051792AA (OR = 2.48; 95% CI: 1.23-4.98, *P* = 0.011) to be significant risk factors for HCC.

## DISCUSSION

This study examined HLA alleles and KIR genes in Japanese patients with chronic hepatitis C for associations with HCC development and revealed significant relationships for HLA-Bw and -C alleles as well as combinations of KIR-HLA in chronic HCV infection. Moreover, a KIR-HLA and SNP in the MICA gene were significantly associated with earlier development of HCC at a cut-off age of 65 years.

Regarding the role of class I HLA-restricted T-cell immune responses in the control of HCV pathogenesis [23], several studies have uncovered associations between HCV infection and HLA class I alleles [19, 24]. Here, HLA-Bw4 was significantly more frequent in patients with chronic HCV infection than in healthy subjects. Conversely, subjects with chronic hepatitis C had a significantly lower frequency of HLA-C2. It has been well documented that certain combinations of KIR and KIR ligands are associated with susceptibility to HCV infection. This study revealed that patients with chronic hepatitis C had a significantly higher incidence rate of KIR3DL1-HLA-Bw4 pairs. It was described elsewhere that individuals carrying KIR3DL1-HLA-Bw4 were of higher NK cell functional potential than HLA-Bw4-negative individuals since the KIR3DL1+ NK subset in HLA-Bw4-positive subjects was more potent [25, 26] and that KIR3DL1 receptors did not bind Bw6 allotypes [27], which was consistent with NK cell licensing [28, 29]. Thöns et al. [30] recently showed that KIR3DL1-HLA-Bw 80(T) was associated with spontaneous clearance of HCV infection in intravenous drug users.

Peripheral and intrahepatic NK cells in HCC patients were reported to display impairments in cytotoxicity and interferon-γ production [31]. Wu et al. described that the accumulation of functional NK cells in HCC tissues could predict improved patient survival, while NK cells were decreased in number, with impaired tumor necrosis factor-α and interferon-γ production, in patients with advanced-stage HCC [32]. HCV-induced HCC is a multistep, progressive liver disease likely influenced by both environmental and genetic factors. Given the functional mechanism and extensive genomic diversity of KIRs and their HLA ligands, several specific KIR-ligand combinations could accelerate disease progression. In fact, the combination of KIR3DS1 and HLA-Bw4 or -Bw4(I80) was under-represented in HCC patients as compared with HCV carriers without HCC from Spain and Italy [20, 21]. However, our data did not confirm these results or show associations among HLA alleles, KIR genes, or KIR-HLA pairs and HCV-related HCC.

The prevalence of older patients with HCC has been increasing in Japan [2]. which is also an impending problem in other countries with recent viral spread. Asahina et al. reported that elderly patients of ≥65 years were at higher risk for HCV-related HCC and that aging was a risk factor for HCC [33]. This prompted us to analyze whether KIR-HLA combinations influenced the development of HCV-related HCC in this age group. Interestingly, we revealed associations between KIR2DL2-HLA-C1 and KIR2DS2-HLA-C1 combinations and HCC in younger individuals. Aging is associated with changes in the frequency, phenotype, and function of NK cell subsets [34]. Lutz et al. revealed an age-related decrease in CD94 and NKG2A expression and a reciprocal age-dependent increase in KIR expression, although this has remained controversial [35]. Moreover, changes in NK cell phenotype in the elderly lead to alterations in cell function, with a decrease in cytotoxicity at the single-cell level and a reduced ability to respond and produce cytokines after stimulation. In the present study, HLA frequencies did not differ between younger and older patients without HCC. Therefore, specific KIR-HLA pairs are presumed to participate in immunosurveillance for HCC. The frequency of KIR2DL2-HLA-C1 was significantly higher in younger patients with HCC (28.8% versus 16.5%; *P* = 0.039; OR = 2.05) when KIR-HLA combinations were examined between younger patients with HCC and those without. There were no significant differences between older patients with and without HCC. To explain the higher frequency of KIR2DL2-HLA-C1 in younger HCC patients, we considered that NK-cell activation was inhibited by KIR2DL1-HLA-C1, thus rendering them unable to kill HCC cells. However, such a notion is highly speculative and requires additional studies to validate. At present, we can only describe a possible relationship of KIR2DL2-HLA-C1 with HCC development in younger patients.

A recent genome-wide association study revealed that rs2596542 in the 5′ flanking region of MICA was associated with HCV-related HCC and soluble form MICA levels in the Japanese population [22]. Furthermore, the serum soluble form of MICA was elevated in advanced HCC patients and associated with downregulated NKG2D expression and impaired NK cell activation [36]. The engagement of MICA and NKG2D strongly activates NK cells, enhancing both cytotoxic granule exocytosis and cytokine secretion. [37] NKG2D ligand (MICA or MICB) recognition by NKG2D induces and/or enhances immune responses to cancer cells [38]. Hence, the pathophysiological effects of MICA SNPs represent a prognostic biomarker for patients with chronic hepatitis C-induced HCC [39]. Although our findings did not support a relationship of SNPs in the MICA gene with HCC, they did reveal associations with HCC in younger patients. The reasons for this discrepancy may be attributed to the smaller number of patients in the present study.

In logistic regression analysis of our cohort, KIR2DL2-HLA-C1C1 and rs1051792AA in the MICA gene were independent risk factors related to HCC in patients of less than 65 years, indicating at least two genetic factors that may offer clinicians additional insights into predicting HCC development in younger individuals. Kaplan-Meier analysis of HCC development in a non-HCC group over time would be an ideal method to clarify whether our identified genetic factors imparted susceptibility to HCC development. Since the present study is cross-sectional, further large-scale prospective investigations are needed to confirm our findings.

In conclusion, HLA alleles, KIR3DL1-HLA-Bw4, and KIR2DL1-HLA-C2 are associated with either HCV susceptibility or protection in the Japanese population. KIR2DL2-HLA-C1 and KIR2DS2-HLA-C1 are novel KIR-HLA pairs linked to the development of HCC in younger patients, as are SNPs in the MICA gene. Investigation of the frequency, phenotype, and function of NK cells in age-stratified patients with HCV-induced HCC will be necessary to identify the precise mechanism of susceptibility to HCC in Japan.

## MATERIALS AND METHODS

### Study population

A total of 787 consecutive patients with chronic hepatitis C were enrolled in this retrospective study from Shinshu University Hospital in Matsumoto, Japan, between September 2003 and August 2015. We also recruited 325 volunteer control subjects from hospital staff who had indicated the absence of any major illness in a standard questionnaire. The racial background of all patients was Japanese. Eligible patients were at least 20 years of age and confirmed to have chronic hepatitis C based on the previously reported criteria of the presence of serum anti-HCV antibodies and detectable HCV RNA [40]. All patients and controls were negative for the hepatitis B surface antigen and antibodies to the human immunodeficiency virus. Patients who exhibited other causes of chronic liver disease, such as alcoholic liver disease, non-alcoholic liver disease, primary biliary cholangitis, or autoimmune hepatitis, were excluded. Serum levels of HCV RNA were determined using the Cobas TaqMan HCV test (Roche Diagnostic Systems, Tokyo, Japan) with a linear dynamic range of 1.2-7.8 log IU/mL. HCV genotypes were determined by sequence analysis. Total bilirubin, alanine aminotransferase, and other relevant biochemical tests were performed using standard methods. The diagnosis of HCC was based on hypervascularity confirmed by dynamic computed tomography and/or magnetic resonance imaging when serum alpha fetoprotein level was increased or a mass lesion was detected by ultrasonography. This study was conducted in accordance with the principles of the 2013 Declaration of Helsinki after review and approval by the ethics committee of Shinshu University School of Medicine in Matsumoto, Japan (No. 527). Informed consent was obtained from each patient included in the study.

### HLA class I, KIR, and MICA genotyping

Genomic DNA from patients and controls was isolated from whole blood samples using QuickGene-800 assays (Fujifilm, Tokyo, Japan). HLA-Bw4, -C1, and -C2 genotyping [41] and KIR genotyping [42] were performed using PCR with sequence-specific primers. HLA typing was combined with KIR typing to stratify patients according to predicted KIR-ligand interactions and binding affinities. These KIR-HLA pairs were KIR2DL1/2DS1-HLA-C2, 2DL2/3/2DS2-HLA-C1, and 3DL1/3DS1-HLA-Bw4. Genotyping of rs2596542 and rs1051792 in the MICA gene was performed using the TaqMan SNP genotyping allelic discrimination method (Applied Biosystems, Foster City, CA). The rs2596542 has been associated with HCV-induced HCC in a GWAS [22], and rs1051792 corresponds to a methionine/valine polymorphism at amino acid 129 of MICA, with the methionine allele as a strong binder to NKG2D [43]. Both SNPs had minor allele frequencies of >5%. All genotyping was blinded to clinical variables.

### Statistical analysis

The Mann-Whitney U test was employed to analyze continuous variables. Pearson's chi-squared test or Fisher's exact test was used for the analysis of categorical data. A *P* value of <0.05 was considered to be statistically significant. Association strength was estimated by calculating the odds ratio (OR) and 95% confidence interval (CI). Stepwise logistic regression analysis with a forward approach was performed to identify independent factors associated with HCC that were separated into two categorical variables by their median value. Variables associated with a *P* <0.05 in univariate analysis were included in this step. Statistical analyses were carried out using SPSS Statistics 24 software (IBM, Tokyo, Japan).

## Abbreviations

HCC: hepatocellular carcinoma
HCV: hepatitis C virus
HLA: human leukocyte antigen
KIR: killer cell immunoglobulin-like receptor
MHC: major histocompatibility complex
MIC: major histocompatibility complex class I chain-related gene
NK cell: natural killer cell
OR: odds ratio
SNP: single nucleotide polymorphism
